# Supplementary material for: Using electronic medical records to understand the impact of SARS-CoV-2 lockdown measures on maternal and neonatal outcomes in Kampala, Uganda
Source: PLOS Glob Public Health. 2023 Dec 8;3(12):e0002022. doi: 10.1371/journal.pgph.0002022 (PMC10707482; doi:10.1371/journal.pgph.0002022)
Supplement: S1 Table — (PDF) [file pgph.0002022.s001.pdf]

S1 Table. Level of Government Restrictions on the spread of COVID

Phase Code	Time period	Phase/level of restrictions in place	National restrictions	Hospital level restrictions	COVID-19 in Africa
1	01st Jan 2020 - 17th Mar 2020	Pre-lockdown period, international outbreak—No restrictions but warnings to the public	Nil	Nil	14th February: First case in Africa (Egypt)<br><br>28th Feburary:<br>First case in sub-Saharan Africa (Nigeria)<br><br>13th March:<br>First case in East Africa (Kenya)
2	18th Mar 2020 - 25th May 2020	Partial Lockdown and Complete/Total lock down and	18th March:<br>32 days suspension of mass gatherings.<br>Mandatory quarantine on arrival in country (14 days)<br><br>20th March:<br>Schools closed for 30 days<br><br>21st March:<br>Borders closed except for cargo and goods<br><br>25th March:<br>Public transport suspended for 14 days and restrictions placed on private vehicle movements.<br><br>30th March:<br>Nationwide curfew announced: 7pm to 6:30am for 14 days	23rd March to 21st April, 2020<br>Complete closure of all antenatal, sexual health and vaccination services	21st March 2020:<br>Uganda reports first case

			10th April:<br>Mandatory testing of truck drivers<br><br>12th April:<br>Lockdown and curfew extended for further 21 days<br><br>4th May:<br>Lockdown and curfew extended. Face masks in public declared mandatory.<br><br>18th May:<br>Lockdown extended		6th May: Uganda reaches 100 confirmed cases of COVID-19<br><br>18th May: Uganda reaches 200 confirmed cases of COVID-19
3	26th May 2020 - 06th Jun 2021	Post lock-down period with minimal restrictions on movement	26th May 2020:<br>Easing of restrictions: private cars with 3 people and general merchandise shops allowed with strict social distancing<br><br>4th June 2020:<br>Public transport in non-border districts resumes at half capacity		09th June :<br>Over 1000 confirmed COVID-19 cases<br><br>23rd July 2020<br>Uganda reports 1st death from COVID-19
4	07th Jun 2021 - 31st Jul, 2021	Second Lock down, less restrictive compared to the 1st lockdown	June 7th 2021:<br>School closures, suspension of inter-district travel, markets closed, suspension of church services, restrictions on public transport		
5	01st Aug 2021 – 30th Oct 2021	Post lock-down, limited restrictions	July 31st 2021<br>Public transport resumes with half occupancy. Private vehicles allowed 3 occupants.<br><br>September 22nd 2021<br>Social venues, church services can re-open.<br>Bars remain closed<br>Curfew of 7pm maintained.		
