# Supplementary material for: Using electronic medical records to understand the impact of SARS-CoV-2 lockdown measures on maternal and neonatal outcomes in Kampala, Uganda
Source: PLOS Glob Public Health. 2023 Dec 8;3(12):e0002022. doi: 10.1371/journal.pgph.0002022 (PMC10707482; doi:10.1371/journal.pgph.0002022)
Supplement: S2 Table — (PDF) [file pgph.0002022.s002.pdf]

S2 Table. Missing data pattern and model used

Variable	Obs	Missing	Percent missing	Model used
openmrsid	27980	0	-	N/A
age	27980	6	0.0	Predictive mean matching
gravida	27980	3343	12.0	Proportional odds regression model
parity	27980	2159	7.7	Proportional odds regression model
Weeks of gestation	27980	4245	15.2	Predictive mean matching
Mode of Delivery	27980	2882	10.3	Polytomous regression
Apgar Score at 1 minute	27980	4410	15.8	Proportional odds regression model
Apgar score at 5 minutes	27980	4258	15.2	Proportional odds regression model
Baby Birth weight	27980	3601	12.9	Predictive mean matching
Delivery Outcome	27980	7545	27.0	Polytomous regression
Condition of Baby at Discharge	27980	2895	10.4	Polytomous regression
Obstetric Diagnosis	27980	4280	15.3	Polytomous regression
Condition of mother at discharge	27980	2793	10.0	Logistic regression
Any obstetric complication	27980	0	-	N/A
Data of admission	27980	0	-	N/A
