# Supplementary material for: Using electronic medical records to understand the impact of SARS-CoV-2 lockdown measures on maternal and neonatal outcomes in Kampala, Uganda
Source: PLOS Glob Public Health. 2023 Dec 8;3(12):e0002022. doi: 10.1371/journal.pgph.0002022 (PMC10707482; doi:10.1371/journal.pgph.0002022)
Supplement: Attachment — Submitted filename: Reviewer Observation.docx [file pgph.0002022.s003.docx]

Reviewer Observation

1. The aim of the research needs to be clarified and linked with the conclusion and Discussion
2. The first paragraphs of the methodology seem like an introduction, so it is better to include how the system works and its function in the introduction section rather than the method.
3. The table is well generated and presented, but its description parts are low. It is better to explain its finding.
4. We need to clarify the methodology clearly along with research ethics. Informtion is used from the government record system, and how to take consent from each participant needs to be explained more
5. In conclusion, **There is an urgent need for health care systems to plan for future pandemics or 301 periods of insecurity in a way which limits indirect harm to vulnerable populations. Use of 302 EHR and easy to use dashboards may enable healthcare providers and policy makers to 303 rapidly identify at risk populations and consider health care interventions to improve health 304 outcomes in this and future outbreaks and pandemics.** It is written, but how it came is explained in the finding section. Only the finding section describes the status of the mother and child with services.
6. Language needs to review and checked before published
