# Supplementary material for: Using electronic medical records to understand the impact of SARS-CoV-2 lockdown measures on maternal and neonatal outcomes in Kampala, Uganda
Source: PLOS Glob Public Health. 2023 Dec 8;3(12):e0002022. doi: 10.1371/journal.pgph.0002022 (PMC10707482; doi:10.1371/journal.pgph.0002022)
Supplement: Attachment — Submitted filename: Response to Reviewers.docx [file pgph.0002022.s004.docx]

Reviewer comments and response

| **Section** | **Section and Reviewer Comments** | **Response** |
| --- | --- | --- |
| **General Comments** | Reviewer #2: Language needs to review and checked before published | Thank you, we have checked language throughout the manuscript |
|  | Reviewer #4: - Suggest following the STROBE guidelines for observational studies | We have used the STROBE guidelines and included the checklist as an appendix in our revised manuscript. Page 6, line 109-110 |
|  | Reviewer #4: - Consistency in the use of the word "COVID-19"; some parts as COVID19 and some as COVID-19 | This has been amended throughout the manuscript |
|  |  |  |
|  | Review # 1: -Can the author clarify the primary outcome in the abstract? | Primary outcome added/clarified (Page 2, lines 29-31 and lines 32-34) |
|  |  |  |
| **Abstract** | Review # 3- Lines 38-40, authors reported the crude ORs for pre- versus lockdown not pre- versus post-lockdown of preterm and obstetric complications (Table 2). “Comparison of pre-and post-lockdown variables showed an increased risk of preterm birth (adjusted Odds Ratio (aOR) 1.34, 95% CI 1.20-1.49); obstetric complications (aOR 2.63, 95% CI 2.39-2.90)”  -What is meant obstetric complications? | Thank you for noting this error, we have corrected the aOR to pre- vs post-lockdown in the abstract.  Additional description of obstetric complication has been added in the methods section (Page 6, lines 116-120) |
|  | Reviewer #4: Any abbreviation should be defined initially prior to its use (abstract) | Done |
|  |  |  |
|  | **Introduction** |  |
|  | Reviewer #3: -Can you provide the study hypothesis of the study | Hypothesis added (Page 5, lines 84-86) |
|  | Reviewer #2: The aim of the research needs to be clarified and linked with the conclusion and Discussion | We have added our hypothesis and aims on page 5 lines 84-91 |
|  | Reviewer #4: - Objective is not outlined. It is confusing to the reader if this study's primary objective is to demonstrate the use of electronic medical records or examine the pregnancy outcomes as affected by the pandemic. | Primary objective was to demonstrate use of the Electronic medical records (Page 5, lines 86-87) while the secondary objective was to build risk factor model and visualization dashboard (Page 5; lines 87-91) |
|  |  |  |
| **Methodology** | Reviewer #2: We need to clarify the methodology clearly along with research ethics. Information is used from the government record system, and how to take consent from each participant needs to be explained more | Information on informed consent added page 9-10 lines 192-199 |
|  | Reviewer #2: The first paragraphs of the methodology seem like an introduction, so it is better to include how the system works and its function in the introduction section rather than the method. | This information has been moved to introduction section, Page 4-5 lines 66-83 |
|  | Reviewer #3: -Lines 102-103, authors stated that “mode of delivery (defined as 1-born before arrival, 2-born by caesarean section, or 3-born by spontaneous vaginal delivery)” what about asisted vaginal delivery? Were they removed from the analysis? | We have corrected the text to reflect information in the tables and added more explanation on this variable (i.e. mode of delivery has been replaced with obstetric diagnosis) page 6 lines 115-116. Assisted delivery was not a valid data option in the MoH tools used for data collection |
|  | Reviewer #3 -Line 10 what is meant by obstetric complications, clarify this outcome refers to what exactly?  Reviewer #4- The variables need to be clearly defined (i.e., how is obstetric complication defined?) | We have added a definition and more explanation of Obstetric complication, Page 6, lines 116-119 |
|  | Reviewer #3-How did the author specify whether the adverse outcomes were due the lockdown or pandemic itself? Are data available on COVID-19 for the mother? Was this consider in the analysis stage? | Data for the mother on COVID19 was not available for this analysis. We however compared proportion of the adverse outcomes pre-, during and post-pandemic. (Page 7, lines 139-140) |
|  | Reviewer #3: -Regarding missingness, there is a substantial amount of missing data on preterm delivery (52%) in the Pre-Lockdown stage, imputation might be not suitable for this variable.  Reviewer #3-More than 50% of data on preterm are missing in the pre-lockdown stage, were those with missingness excluded from the analysis in Table 2? | Our assessment of missingness for the independent variables was done to facilitate the selection of variables to be included in the analysis. We included only variables with <30% overall missingness (S1 table). In our case “Delivery outcome” had overall, 27% missingness, although it had 52% missingness in the Pre-lock period (Page 8, lines 159-165). We have added additional explanation in the methods Page 8, line 154-160 |
|  |  |  |
| **Results** | Reviewer #2: The table is well generated and presented, but its description parts are low. It is better to explain its finding. | Table 1 has been revised to present only the demographic characteristics and descriptions added accordingly. Page 11 |
|  | Reviewer #3: -The authors might consider calculating the difference between the change in each outcome from the prelockdown to postlockdown periods | Given that our outcomes were categorical, calculating change in them is generally meaningless If the reviewer feels strongly that we should undertake this analysis, we can add the supplemental table |
|  | Reviewer #3: -I could not read data on Figure 1 & 2, may be consider increasing the resolution of them.  -Also Table S2 was not available with the submitted materials | Clearer Figures 1&2 have been redone  We can confirm that Table S2 was uploaded |
|  | Reviewer #3: -Lines 195-195 “There were higher proportion of children born at gestational age <32 weeks; higher proportion of children with lower APGAR score (<7) at 1 and 5 minutes; higher proportions women with obstetric complications; higher preterm births during and after COVID outbreak compared to the period before COVID outbreak, Table 1” I disagree with these interpretations, given the number of missingness on gestational age, APGAR score, you cannot confirm that there I high proportion of these outcome before after/during lockdown. | Interpretation of results in table 1 have been revised (Pages 10-11, lines 215-219). We have also added the missingness as a limitation in our discussion section |
|  | Reviewer #3: -Lines 197-198, ‘’ during and after COVID outbreak compared to the period before COVID outbreak” do you mean during and after COVID lockdown and before the lockdown period? -Line199, correct COVI09 to COVID19 | This has been clarified throughout the manuscript |
|  | Reviewer #3-Line 219 “The odds of preterm birth were highest in younger mothers (age<20, aOR 1.14-1.40) compared to mothers aged 25-34 year” you write the CI, add the aOR=1.26.  Reviewer #3_can the authors be consistent when reporting the finding, sometimes the lockdown period refers to as before the outbreak COVID 19.  Reviewer #3-There are discrepancies between reported data in the text and that from the Table. In lines 245-247, “The odds of undergoing caesarian section were higher in younger mothers (age<20 years, aOR 2.48, 1.96-3.15 and those aged 20-24 years aOR 1.80, 1.59-2.03) compared to mothers aged 25-34 years while it was lower among older mothers aged 35+years (aOR: 0.66, 055-0.79).” author reported data related to born before arrival as data on CS (Table 3), correct these data in the text. | This has been corrected. Page 12, lines 237-239  This has been corrected throughout the manuscript  This has been corrected. Page 13, lines 273-27 |
|  | Reviewer #3- Study design is not presented in the methods section  Reviewer #4- The p-values for table 1 should be listed | Our study is a secondary data analysis of existing EHR data. Study design is cross-sectional panel data study (page 7, line 126-130)  We have amended the table and added the p-values to table 1. Page 11 |
|  |  |  |
| **Discussions** | Reviewer #2: - Without having a clear understanding of the objective/aim of the study, it was difficulty to analyze the discussion and conclusion sections | Aims and objectives of the study have been clarified (Page 4-5, lines 84-91) and we have rewritten the discussion linking it well with the aims and objectives of the study. |
|  | Reviewer #2: - The impact of shutdown need to be addressed in the manuscript (for instance, was there an influx of patients admitted to the ICU/deployment to critical care services that would impact the care rendered to obstetric patients) | We have added the Impact of shutdown due to COVID-19 (Page 17, lines 308-315). Table S1 provides an overview of lockdown restrictions. |
|  | Reviewer #3: -The findings of the current study should be compared with that of the previous studies. | Our discussion has now been expanded, highlighting similarity of findings from our study with previous studies page 17-19, lines 299-364 |
|  | Reviewer #3: -The authors should highlight the limitations in the discussion, in particular, unavailable data on important risk factors, such as hypertensive disorder during pregnancy, DM covid-19 and. -Also, the application and direction for further studies should be discussed | We have added limitations in respect to unavailable data on hypertension disorder during pregnancy (Page 18-19, lines 346-352)  Areas for further research added (Page 19 lines 353-356) |
|  |  |  |
| **Conclusions** | Reviewer #2: In conclusion, **There is an urgent need for health care systems to plan for future pandemics or 301 periods of insecurity in a way which limits indirect harm to vulnerable populations. Use of 302 EHR and easy to use dashboards may enable healthcare providers and policy makers to 303 rapidly identify at risk populations and consider health care interventions to improve health 304 outcomes in this and future outbreaks and pandemics.** It is written, but how it came is explained in the finding section. Only the finding section describes the status of the mother and child with services.  - Conclusion and recommendation should be linked with the finding section. | The aim and goals of the study have been added. We have also revised the discussion and conclusion and this now links well with the goals and objectives of the study. We thank the reviewer for their useful input. |
|  |  |  |
| **References** | Reviewer #3- The authors started with reference #7 in the introduction, what about reference from 1 to 6?  -References 11 & 12 are incomplete and irrelevant to the statements. | All references have been updated (Page 21-22) |
