# Supplementary material for: Using electronic medical records to understand the impact of SARS-CoV-2 lockdown measures on maternal and neonatal outcomes in Kampala, Uganda
Source: PLOS Glob Public Health. 2023 Dec 8;3(12):e0002022. doi: 10.1371/journal.pgph.0002022 (PMC10707482; doi:10.1371/journal.pgph.0002022)
Supplement: Attachment — Submitted filename: comment-2.docx [file pgph.0002022.s005.docx]

Comment

1. Information is well presented, and the author tried to review the manuscript based on comments.
2. Stil the title and conclusion are not matched
3. Methodology and analysis describe more about the importance of the records system but not the finding and analysis.
4. The analysis table is good but does not explain it and make a conclusion.
5. What will be the recommendation of this research, and what is the way forward
6. What is explained about the dashboard, and why is it essential in the study
7. Data is analysed on the electronic record system, and how they get informed consent where recoding information was taken but not explained about the data analysis and presentation.
