# Supplementary material for: Using electronic medical records to understand the impact of SARS-CoV-2 lockdown measures on maternal and neonatal outcomes in Kampala, Uganda
Source: PLOS Glob Public Health. 2023 Dec 8;3(12):e0002022. doi: 10.1371/journal.pgph.0002022 (PMC10707482; doi:10.1371/journal.pgph.0002022)
Supplement: Attachment — Submitted filename: REBUTTAL LETTER.docx [file pgph.0002022.s006.docx]

Dear reviewers,

We thank you for your time and attention in reviewing our submission to PLOS Global Health. We have taken these comments into account and made changes to our manuscript. Responses by reviewer comment can be seen below.

Best wishes,

Dr. Lauren Hookham

|  | Comment | Author response |
| --- | --- | --- |
| Reviewer 2 | title and conclusion are not matched | Changed to:  **Using electronic medical records to understand the impact of SARS-CoV-2 lockdown measures on maternal and neonatal outcomes in Kampala, Uganda** |
|  | Methodology and analysis describe more about the importance of the records system but not the finding and analysis. | The focus of the manuscript is to demonstrate the utility of data synthesis for decision making. We have added additional paragraphs (from line 303) to discuss this in more detail. |
|  | The analysis table is good but does not explain it and make a conclusion. | Table has title and explanation Additional commentary in discussion section added (as per point above). |
|  | What will be the recommendation of this research, and what is the way forward | Added  “Electronic health records can be utilised to rapidly synthesise data in periods of rapid policy intervention to assess indirect effects and should be considered as a key tool for health monitoring.”  Line 404 - 409 |
|  | What is explained about the dashboard, and why is it essential in the study | We have added additional detail from line 406 – 409 |
|  | Data is analysed on the electronic record system, and how they get informed consent where recoding information was taken but not explained about the data analysis and presentation. | The consent process is reported from line 220 – 228. Data was extracted from an anonymous Ministry of Health data records system. No additional consent was needed bar what is reported. |
| Reviewer 3 | Dr. Ouma and colleagues present a revised version of their manuscript titled " Using routine data to understand adverse pregnancy and neonatal outcomes associated with the COVID-19 pandemic in Kampala, Uganda." The authors respond to some of the previous critiques. Key edits include the following: correcting the reported aOR in the abstract. Previous version did not include a hypothesis, the authors now included their hypotheses at the conclusion of the introduction. The authors added the limitation of not considering other comorbidities (potential confounders) on the reported association. The authors also clarify the meaning of obstetric complications | Thank you |
|  | If data not available of whether a delivery was assisted or not, then I suggest to change SVD to VD as we do not know whether it is a spontaneous or not. | There were no assisted deliveries – only SVD or caesarian section, this has been clarified in the methods |
|  | Clarify any abbreviation in the Tables at the footnote, such as that in Table 2 | This has been corrected. |
|  | Line 217, remove “; most of the women were multi-gravida.)”. | This has been corrected. |
|  | In table 1, authors should provide the frequency and percentage of missing data in the related categories, so reader can be clear about the proportion of missing data in each group. | We have added the total number of missing records overall to enable the reviewer to better interpret the data and have added this as a limitation (line 416-417). |
|  | Lines 218-219, “There were more preterm births during and after COVID-19 lock-down period compared to the pre-lock down period; Table 1.”. I still disagree with this interpretation and I think this is misleading statement as more than half of data in the pre-lockdown are missing according to the Table in the first version of the manuscript | The authors accept that missing data limits interpretability of this statement but we still feel that this highlights a change in outcomes that is worthy of discussion. However, we have now removed this statement from the manuscript to allow readers to draw their own conclusions on the data without overrepresenting our findings |
|  | Lines 349 -351 “Other limitations include the unavailability of important risk factors such as pre-existing diabetes 350 mellitus, hypertension, and cardiovascular diseases which are important risk factors for COVID-19 disease.” Correct that they are important risk factors for adverse maternal and pregnancy outcomes. | This has been corrected. |
|  | Some references require editing. | This has been corrected. |
